# Anger and Post-Traumatic Stress Symptoms in Firefighters After a Firefighting Operation with Two Team Member Fatalities

**DOI:** 10.3390/healthcare13192395

**Published:** 2025-09-23

**Authors:** Tabea Görlich, Vanessa Borck, Nils Hüttermann, Francesco Pahnke, Tristan Wellendorff, Ulrich Wesemann

**Affiliations:** Department of Psychiatry, Psychotherapy and Psychotraumatology, Bundeswehr Hospital Berlin, Scharnhorststr. 13, 10115 Berlin, Germany

**Keywords:** aggression, anger, anxiety, disaster, fatalities, firefighters, PTSD

## Abstract

Due to the burdens of critical operations, firefighters are at occupational risk of developing mental health problems such as post-traumatic stress symptoms (PTSS) or anger. In this study, we assessed the impact of a firefighting operation with two fatalities and eleven injuries among colleagues on the mental health of those involved. **Aims:** The study hypothesizes that firefighters who were acquainted with the wounded or deceased show more PTSS and score higher on the STAXI-2 State Anger, Anger Expression-In/Out, and Anger Control-In/Out compared to those who were not. The second hypothesis assumes that the deployed group shows more PTSS and scores higher on the STAXI-2 State Anger, Anger Expression-In/Out, and Anger Control-In/Out compared to the grouping of non-deployed colleagues. **Methods:** A total of N = 138 firefighters were included, of which n = 32 were deployed and n = 106 were not (n = 26 acquainted with wounded or injured; n = 107 not acquainted with wounded or injured; n = 5 not specified). Both groups completed a standardized questionnaire voluntarily and after providing written informed consent ten months after the incident. The questionnaire consisted of the Posttraumatic Stress Checklist for DSM-5 (PCL-5) and the State-Trait Anger Inventory (STAXI-2) questionnaire. **Results:** Firefighters who knew the injured or deceased colleagues had significantly higher PTSS [T(23.7) = −2.5; *p* = 0.019; d = 0.67; 95% CI = (−10.8, −1.0)], state anger [T(74.2) = 2.4; *p* = 0.021; d = 0.41; 95% CI = (0.2, 2.0)], and anger control scores [T(124) = −2.7; *p* = 0.008; d = 0.71; 95% CI = (−5.9, −0.9)]. In addition, the deployed group showed higher anger control [T(134) = 2.0; *p* = 0.046; d = 0.42; 95% CI = (0.0, 4.2)] and outward anger expression [T(73.5) = −2.2; *p* = 0.032; d = 0.40; 95% CI = (−2.3, −0.1)] scores compared to their non-deployed colleagues. **Discussion:** Future psychological pre- and post-deployment interventions should place more focus on the increased vulnerability of those acquainted with wounded or deceased colleagues.

## 1. Introduction

Emergency responders are likely to face tasks such as caring for the injured, recovering the deceased, and situations where they find their own safety to be at risk [1]. Such experiences make them vulnerable to psychological impacts such as post-traumatic stress disorder (PTSD) or anger [2,3,4]. PTSD describes a mental health condition that is caused by a distressing life event. It is defined by post-traumatic stress symptoms (PTSS) such as reexperiencing, avoidance, negative alterations in cognition and mood, and hyperarousal that last for more than a month [5]. The presence of a PTSS does not automatically lead to a PTSD diagnosis.

Recent data shows that engagement in routine duties showed a globally pooled PTSD prevalence of 14.3% in first responders. Furthermore, a PTSD prevalence of 8.3% was recorded following large-scale disasters. Among the group of first responders, firefighters showed a PTSD prevalence of 12.1% related to routine duties and 9.0% in relation to large-scale disasters [6]. A scoping review that explored trauma and mental health among first responders found that firefighters reported the highest rates of PTSD among first responders [7]. Among German volunteer firefighters, a 2020 study reported a PTSS prevalence of 12.5% [8], while an earlier study showed a PTSS prevalence of 18.2% in German professional firefighters [9].

Anger has been found to be associated with PTSD [10]. Moreover, a 2022 study including individuals with PTSD observed suppressed anger to be more prevalent in first responders than in civilians [11]. Among firefighters, anger might be a relevant factor for predicting the severity of comorbidities such as PTSD, depression, alcohol use, and symptoms of insomnia [12,13,14]. These comorbidities have been reported to be more prevalent among firefighters than the general population [6,7,15,16,17,18]. Therefore, anger symptoms should be of special interest when studying firefighters’ mental health after distressing incidents to predict and eventually prevent common comorbidities.

Although work-related mental health burdens on firefighters have been documented in previous studies, only a few address the impact of singular potentially distressing events. Such events could be identified in an earlier study conducted among US-American firefighters. When asked to name their single worst work-related event, they named the death of an adult, the death of a child, and the death of a colleague [19]. Firefighting operations that lead to experiencing the death of a child or the death of a colleague are especially rare (18 cases of voluntary firefighter deaths during an operation in Germany between 2015 and 2020) [20,21]. This limits the current available data concerning firefighters’ mental health burdens following distressing work-related events even further.

The objective of this study is to improve on the current research gap surrounding the impact of the on-duty death of firefighters on their colleagues. In this context, we assess the mental health burdens among firefighters after the deaths of two colleagues during a firefighting operation near the city of Bonn. The study focuses on the participants’ deployment during the operation and their acquaintance with the wounded or deceased since these factors had previously been reported to have a high impact on first responders [19]. Based on previous findings, we chose to focus our investigation on PTSS and anger symptoms [10,22,23].

To establish our hypotheses, we identified previous studies that explored topics related to the firefighting operation we investigated. We found that firefighters and paramedics had named the witnessed and non-witnessed death of a coworker as their highest-ranking incident stressors in a previous study [24]. The more common occurrence of experiencing work-related violence against oneself or coworkers has since been identified as a major stressor and health risk for emergency responders [25,26]. These findings strengthened our assumption that the death or injury of coworkers had a strong impact on firefighters that were acquainted with the wounded or deceased. We therefore placed a special focus on the impact of a personal acquaintance with the wounded or deceased.

To form a point of reference for the impact of the incident, we differentiated between a deployed group and a non-deployed group. A previous study had shown that higher levels of trauma exposure increased the risk of developing PTSD in volunteer firefighters [27]. This finding supported our decision to differentiate between the two groups. Additionally, we found that previous studies had documented an association between PTSS and anger [10,22,23]. Anger also proved to be a predictor of comorbidities like depression, alcohol use, and insomnia [11,12,13,14]. We therefore decided to place a focus on the topics “PTSS” and “anger”.

The hypotheses of this study were chosen accordingly:

**H1.**  *Firefighters who were acquainted with the wounded or deceased show more PTSS and score higher on the STAXI-2 State Anger, Anger Expression-In/Out, and Anger Control-In/Out compared to those who were not acquainted. We hypothesized possible acquaintance effects to be independent of deployment.*

**H2.**  *We hypothesized for the deployed group to show more PTSS and score higher on the STAXI-2 State Anger, Anger Expression-In/Out, and Anger Control-In/Out compared to the grouping of non-deployed colleagues.*

This study is part of the CASH (Calamities, Anxiety, Stress, Hostility)-Force project. Within this project, we assess the mental health burdens of emergency personnel after especially distressing operations. These events are differentiated according to whether they were caused intentionally and whether they were long- or short-lasting. The firefighting operation considered in this study is an example of a short-lasting event that was not caused intentionally. The long-term goal of the CASH-Force project is to identify add-ons for post-incident interventions [28].

## 2. Methods

### 2.1. Study Approach

The study was conducted in the form of a written survey and centered around a firefighting operation with two team member fatalities that took place on the 18th of June 2023. It employed a Basic Questionnaire to collect socio-demographic data and two standardized questionnaires (PCL-5, STAXI-2) to collect statistical data. The head officials of all fire stations with involvement in the operation were identified and contacted during the first ten months following the incident. Data collection was then conducted twelve months after the incident, with a maximum deviation of two months for mailed questionnaires. This period accounts for possible delayed-onset PTSD and corresponds to the one-year assessment prevalence.

### 2.2. Participation Among Fire Departments

Following the invitation, a total of N = 138 firefighters were included in the study. Among those, n = 32 (23%) were deployed during the operation, and n = 106 (77%) were not. The comparison group consisted of colleagues from the same units who were not deployed due to different working and shift times. In total, the group was made up of 116 (84%) professional firefighters, 13 (9%) voluntary firefighters, and 9 (7%) firefighters that did not specify their affiliation. The city of Bonn counts an estimated 370 professional firefighters, which puts their participation rate at 31%. The voluntary firefighters from the affected municipal district, Sankt Augustin, count 270 members, which puts the participation of voluntary firefighters at 5%.

### 2.3. Informed Consent and Ethical Approval

The assessment of participants’ psychological responses was carried out by using the questionnaire described below, which was completed after an introduction of the study. All participation was voluntary and required informed and written consent.

The ethics committee of the Charité–Universitätsmedizin Berlin approved this study (number: EA4/085/18).

### 2.4. Data Collection Process

The questionnaires were conducted at the participating fire stations after arranging appointments with their respective head officials. They were conducted 12 months after the incident with a two-month time window due to the use of mailed questionnaires. The completion of the questionnaire required approximately 45 min. Every appointment was announced in advance and immediately before the conduction of the questionnaire to allow for planned and spontaneous participation. The participants were taken off-duty during the completion of the questionnaires. Before starting their questionnaire, the participants received a briefing on the study, their voluntary participation, data protection in the form of pseudonymization, and the possible risk of experiencing short-term stress due to engaging with the subject matter. After partaking in the briefing, the participants had the option to complete their questionnaires immediately or to complete them during the following two months and return them by mail. All participants were made aware of their option to receive consultation as well as treatment at the Department of Psychiatry, Psychotherapy, and Psychotraumatology of the Bundeswehr Hospital Berlin if necessary [28].

### 2.5. Applied Questionnaires

A Basic Questionnaire was applied to obtain participants’ relevant socio-demographic information. The questionnaire functioned as a self-report questionnaire and included demographic questions (age, years of service, gender, employment relationship, rank, relationship status) and dichotomous questions (deployment during the operation, acquaintance with the wounded or deceased).

PTSD was assessed using the Post Traumatic Stress Disorder Checklist for DSM-5 (PCL-5), a 20-item self-report questionnaire with a 5-point scale. The cutoff value for PTSD was set at a score of 33. The PCL-5 explores PTSS such as reexperiencing, avoidance, negative alterations in cognition and mood, and hyperarousal [29].

Anger expression was assessed using the German version of the State-Trait Anxiety Inventory-2 (STAXI-2). It consists of 51 items and functions as a self-report questionnaire, employing a Likert scale of 1–4. By applying STAXI-2, state anger (feelings of anger, verbal anger impulses, and physical anger impulses), trait anger (temperament and reaction), and anger expression (anger expression-in/out and anger control-in/out) were measured [30].

### 2.6. Statistical Evaluation

The statistical evaluation of the questionnaire was conducted with IBM SPSS Statistics for Windows version 21.0 (Armonk, NY, USA). We applied chi-square (χ^2^) tests to compare the participants’ demographic backgrounds. Furthermore, independent-samples *t*-tests with α = 0.05 were used to test differences between the deployed group and the non-deployed group. They were also applied to test the hypotheses concerning the impact of familiarity between the participants and the wounded or deceased and the impact of post-incident interventions. Before conducting *t*-tests, the normal distribution of the samples was checked. No violations were found, which made the application of a Mann–Whitney U test obsolete. The homogeneity of variance was tested using the Levene test. If violations were found, the degrees of freedom were adjusted downwards accordingly. This was indicated by specifying one decimal place. The data were tested for homoscedasticity, and Welch’s correction was applied in cases of unequal variances to account for differing degrees of freedom. A linear regression analysis was used to test the influence of acquaintance, deployment, and their interaction on the PCL-5 and the STAXI-2 results. The participants filled out their questionnaires independently, which led to the questionnaires being only partially completed. Due to this, the group sizes and degrees of freedom differ according to the respective questionnaires’ degree of completion.

## 3. Results

### 3.1. Demographics

To test for group differences, we applied χ^2^-tests and independent sample *t*-tests. The deployed and non-deployed groups did not differ significantly in their age, years of service, rank, or relationship status (Table 1).

To test the two hypotheses, participants were grouped according to their deployment during the operation as well as their acquaintance with the wounded or deceased. Table 2 illustrates the compositions of the respective groups. Five participants (4%) could not be grouped since they did not provide information on their acquaintance to the wounded or deceased.

### 3.2. Personal Acquaintance with Wounded or Deceased

The main hypothesis that participants who personally knew the deceased or wounded were more likely to develop PTSS was tested by applying a *t*-test. The group that knew the wounded or deceased showed significantly more PTSS, as depicted in Table 3. At the point of measurement, n = 1 (3.1%) participant from the deployed group and n = 1 (0.9%) participant from the non-deployed group showed PTSD.

The participants who personally knew the deceased or wounded were also more likely to show a higher impact on their state anger T(74.2) = 2.4; *p* = 0.021; d = 0.41; 95% CI = (0.2, 2.0) and their anger control T(124) = −2.7; *p* = 0.008; d = 0.71; 95% CI = (−5.9, −0.9) compared to the participants who were not acquainted with the deceased or wounded.

There, they showed higher scores for verbal state anger T(76.3) = 2.8; *p* = 0.007; d = 0.49; 95% CI = (0.2, 1.0), outward anger control T(124) = −2.1; *p* = 0.037; d = 0.50; 95% CI = (−2.9, −0.1), and inward anger control T(124) = −2.4; *p* = 0.017; d = 0.65; 95% CI = (−3.5, −0.3).

In relation to anger expression, no statistically significant differences between the deployed and the non-deployed group emerged.

### 3.3. Deployment During the Operation

The deployed group showed more pathological symptoms compared to their non-deployed counterparts. They showed a higher impact on their overall anger control, scoring higher in categories such as the outward anger control (Table 4).

Besides an impacted anger control, the deployed group also showed higher values for their outward anger expression T(73.5) = −2.2; *p* = 0.032; d = 0.40; 95% CI = (−2.3, −0.1) compared to the non-deployed group.

In relation to the PTSS and state anger, no statistically significant differences between the deployed and the non-deployed group emerged.

### 3.4. Interactions of Acquaintance and Deployment

A linear regression analysis was used to test for a statistically significant influence of acquaintance, deployment, and the interaction between both the PCL-5 and STAXI-2 results. The analysis showed a statistically significant influence of deployment and the interaction between deployment and acquaintance on the participants’ outward anger control (Table 5). Aside from this finding, no further statistically significant influences were found.

## 4. Discussion

The studied operation was included in the CASH-Force project due to the rareness of firefighting operations with casualties and their distressing nature for the emergency personnel involved.

### 4.1. Personal Acquaintance with the Wounded or Deceased

#### 4.1.1. Post-Traumatic Stress Symptoms

The major finding of this study is the impact on participants who were acquainted with the wounded or deceased during the fatal operation. The acquainted group showed higher PTSS, such as intrusions, avoidance, negative cognitions, and overall PTSS, compared to the non-acquainted group.

Negative cognitions and avoiding behavior were also reported in a similar study by De Soir et al. [31]. Their study focused on the Ghislenghien pipeline explosion that claimed the lives of 25 people, with five firefighters among the deceased. The firefighters deployed on site described themselves to be unable to speak of the incident, which might correspond with the symptoms of avoidance and negative cognitions. In the study, firefighters also described their relationship to colleagues as “one big family”, which is also a common notion among German firefighters. The close familiarity among colleagues might have added to the severeness of the prevalent symptoms among the firefighters who were acquainted with the wounded or deceased. Previous studies found evidence of secondary traumatic stress (STS) in family members of veterans with PTSD [32,33]. A similar effect among firefighters might therefore be assumed. To prove this correlation, more studies with a focus on STS between colleagues of first responders will have to be conducted. Due to the nature of the operation, during which two firefighters died despite undertaken rescue attempts, the scenario might have led to a perceived failure to prevent the distressing event. This perception could lead to a moral injury (MI) among participants that further increased their risk for long-term adverse outcomes such as PTSS [34]. Although MI and PTSS were found to possibly be associated [34,35], it is important to differentiate them from one another. While PTSD is a mental health condition related to a distressing life event, MI defines the social, biological, and mental health impact on a person following the witnessing, perpetrating, or failing to prevent an act that violates their moral beliefs and expectations [34].

#### 4.1.2. Anger Control

Besides the higher PTSS, the participants who were acquainted with the wounded or deceased also showed higher scores for outward, inward, and overall anger control compared to the non-acquainted group.

The high scores for anger control imply the participants’ inclination towards controlling and containing feelings of anger. High scores for outward anger control imply a strong effort to control and avoid the outward expression of anger. High scores for inward anger control imply a strong effort to calm down and reduce anger by applying relaxation techniques [30]. In a recent study, anger control difficulties were found to be associated with higher levels of psychological distress in traumatically bereaved individuals [36]. Improvement in anger control has also been set as a goal in the treatment of previously deployed veterans [37]. The associated group’s higher scores for anger control could therefore be interpreted as a positive outcome. Increased anger control might be related to the quick deployment of post-incident interventions. For instance, all first responders who were affected by the incident immediately received invitations to partake in five probationary psychotherapy sessions to assess their need for further support [38]. Participating head officials also reported the immediate and extensive support offered by emergency psychological services (EPS) from North Rhine-Westphalia and surrounding federal states. A beneficial aspect of these measures might be assumed. To our best knowledge, higher scores of anger control among first responders who were associated with the wounded or deceased compared to those who were not are a novel finding. To better understand the impacts on anger control after a distressing incident, further studies with a similar focus will have to be conducted.

#### 4.1.3. State Anger

Participants who were acquainted with the wounded or deceased showed higher scores for verbal and overall state anger compared to the non-acquainted group.

High scores for state anger imply increased feelings of tension, irritation, or anger and an activation of the autonomic nervous system. The high scores on verbal state anger imply an increased inclination to externalize anger verbally by screaming or shouting [30]. To our best knowledge, no applicable studies with a focus on state anger following a distressing event have been conducted so far. Moral injury may be used as an applicable model to explain the prevalence of state anger in participants with acquaintance to the wounded or deceased. During the operation, firefighters witnessed three colleagues enter a building as part of a routine operation. After an unexpected increase in heat radiation, one colleague exited the building while immediate rescue attempts were initiated to retrieve the other two colleagues from the building. Several hours after the beginning of the operation, the two colleagues were recovered dead [38]. The sequence of this operation may well have led to MI through feelings of failure to prevent an event that violated the participants’ moral beliefs. An increase in state anger might stem from the experienced MI [34,39]. This study provides no data concerning a possible direction of anger among the participants. This information might give insight into possible bereavement-related anger among the participants who were acquainted with the wounded or deceased.

#### 4.1.4. Post-Traumatic Stress Symptoms and Anger

An association between PTSS and subsequent anger has been documented in a previous study by Orth et al. [10]. Furthermore, changes in anger symptoms in relation to PTSS have been shown in first responders following distressing events [40]. The higher scores in PTSS, state anger, and anger control among participants who were acquainted with the wounded or deceased compared to those who were not reinforce these previous findings. The direct relation of these findings to a singular event offers a practical confirmation of the previously reported high impact of the death of a colleague on first responders [19,24].

### 4.2. Deployment During the Operation

#### 4.2.1. Anger Control

In comparison with the non-deployed group, the deployed group showed higher scores for outward and overall anger control. These group differences in anger control show the deployed group’s strong efforts to control and avoid anger and especially outward expressions of anger [30]. Additionally, the deployment as well as the interacting factors of deployment and acquaintance were shown to have a significant effect on the outward anger control. As argued earlier, an increase in anger control might be seen as a positive outcome following immediate psychological interventions during and after the operation [36,37,38]. This interpretation is strengthened by the statistically significant influence of deployment as well as the interaction between deployment and acquaintance on outward anger control. After the incident, both factors were connected to an immediate offer of five probationary psychotherapy sessions to assess a possible need for further support after the incident [38]. All the while, the findings might also be interpreted as the deployed groups’ increased feelings of needing to control their anger compared to the non-deployed group. Overall, increased anger control among firefighters who were deployed during a distressing operation, compared to those who were not, appears to be a novel finding.

#### 4.2.2. Anger Expression

When comparing the deployed group to the non-deployed group, the deployed group showed higher scores for outward anger expression.

High scores for outward anger expression imply a higher inclination towards expressing anger with aggressive behavior towards other people or objects. This behavior may include criticisms, insults, or physical aggression [30]. A previous study found higher levels of anger expression to be associated with suicidal ideation and behavior among veterans [41]. An elevated anger expression should therefore be taken into special consideration during post-incident interventions. To our best knowledge, no studies explicitly recorded an elevated anger expression as a symptom following the experience of a distressing event during adult life. However, outward anger expression has been recorded to be associated with perceived stress among navy personnel [42]. Due to the undertaken rescue attempts and several hours passing between the beginning of the operation and retrieving their two deceased colleagues, the deployed firefighters are expected to have been under enormous strain. This might explain the deployed groups’ ensuing increased levels of outward anger expression. To validate this finding, the aspect of long-term trajectories of anger expression following a stressful situation would have to be studied. This study indicates that the deployment during a highly stressful operation leads to increased outward anger expression among firefighters.

#### 4.2.3. Anger Without Post-Traumatic Stress Symptoms

The higher prevalence of anger symptoms without an increase in PTSS that could be shown among the deployed compared to the non-deployed group is a novel finding since anger and PTSS are usually found to be related. This finding may be explained by applying the cognitive appraisal theory by Lazarus and Folkman [43]. In these terms, the event might have been primarily appraised as a failure or injustice without being appraised as a threat to oneself since the deployed firefighters were unable to enter the building and only entered it at a later point to recover their deceased colleagues [38]. The secondary appraisal of the situation most likely led to an understanding of being unable to control the outcome for their deceased colleagues while being able to control the surrounding operation. These appraisals might have led to an increased need for coping surrounding feelings of injustice and perceived failure without an increased need for coping surrounding a direct threat against oneself. A previous study showed that of paramedics who experienced a direct threat to themselves, 5.91% suffered from PTSD, whereas the group that experienced an indirect threat showed a smaller PTSD prevalence of 1.37% [44]. Among combat soldiers, an odds ratio of 6.2 for PTSD was found among soldiers who had experienced a life-threatening military incident during deployment in comparison to their colleagues who had not [45]. This further supports the notion that coping was centered more around a possible MI related to feelings of injustice or failure than around PTSD stemming from perceived threats during the operation. Here it is important to mention that, although MI and PTSD can occur simultaneously, they are not inherently related to one another [46].

The operation might have led to failure-based MI, which describes the failure of maintaining a morally charged identity [46]. The German firefighting emblem shows the fire brigade’s core duties: “Save**–**Extinguish**–**Recover–Protect” (“Retten–Löschen–Bergen–Schützen”) [47]. Here, the term “recover” is defined to include recovering property as well as recovering the dead. When relating these duties to the operation, it is possible that the deployed group might have felt that their moral identity as firefighters had suffered due to an inability to save and protect their coworkers. As a symptom of MI [34], anger might have been a coping mechanism that the deployed firefighters unconsciously applied to the situation.

Due to its novelty, this finding can only be interpreted in a theoretical context. Overall, the prevalence of an impacted anger expression and anger control after a critical incident should raise concerns since anger has been found to be an important factor for the assessment of alcohol use among firefighters [4]. For future studies, we recommend the investigation of interactions between PTSD, MI, and anger following distressing life events.

#### 4.2.4. Building Resilience and Supporting Mental Health

In a scoping review, Smith et al. found that to support first responders’ mental health, it is necessary to build resilience and break down stigma surrounding the subject [48]. Resilience could be strengthened by including realistic simulations of distressing duty-related operations in first responders’ training. Among soldiers, such training has shown beneficial effects on PTSD and stress in two similar studies [49,50]. The training included education on stress control, communication, coping skills, and a realistic simulation and was implemented before military deployment. Participants who completed the simulations before being deployed showed significantly fewer symptoms of stress and PTSD after deployment compared to the control group [49,50]. Although this training was tested on military personnel, a similar effect might be expected in first responders. We therefore recommend including training that emphasizes education on operational mental health as well as realistic simulations of potentially distressing work-related scenarios into first responders’ working lives. Further, it is recommended for emergency service organizations to promote well-being as an integral part of their employees’ everyday working lives. This is supposed to reduce stigma surrounding mental health and allow for the organizations as well as the first responders to reach out to one another to offer or receive help when needed [48]. Peer support among firefighters has also proven to act as a protective factor against the development of emotional damage, alcohol-related problems, PTSD, and suicide risk after experiencing the death of a colleague [51]. Aside from creating a more open approach to mental health between organizations and their employees, the emotional support among first responders plays a crucial role in protecting their mental health.

## 5. Limitations

With a total of N = 138 participants, the study’s general validity could be improved by acquiring a larger number of participants. The results of this study are exclusive to voluntary and professional firefighters. Therefore, they can only be extrapolated for firefighting organizations and organizations with closely related duties. For future studies, it is recommended to compile a more diverse pool of participants, varying in gender and profession, to avoid overlooking possible differences between major subgroups. However, since firefighters in Germany are still predominantly male, the small number of female participants is currently representative of the given circumstances [52]. This study did not account for factors such as the participants’ role during the operation and their exposure intensity. This limits the analysis of the operation’s direct impact on the deployed group. The one-year gap between the incident and the conduction of the study may have affected the severity of PTSS in the participants. A previous study differentiated five different trajectories of PTSD following physical injury over two years. While two of the trajectories showed an increase in PTSD after one year, the other three, including most participants, were all decreasing [53]. Due to the one-year assessment window, this study does not account for a possible initial increase in PTSS after the incident. The accuracy of this study may be affected due to the use of self-report questionnaires instead of an expert rating for data collection. It should also be mentioned that the number of voluntary firefighters in this study might differ slightly, since it is common for professional firefighters to also be part of the voluntary firefighters in Germany. The study does not provide baseline data before the incident, which could be used to further put the results into context. Therefore, confounders such as pre-existing mental health conditions could not be addressed. Due to the voluntary participation in the study, there is a possible selection bias regarding study participation. Firefighters that were more affected by the incident might have felt a higher incentive to join the study.

## 6. Conclusions

Due to the rare nature of first responder operations with casualties among coworkers, this study gives an insight into a very impactful and only sparsely explored topic. It produced two key findings. We found that firefighters who were acquainted with the wounded or deceased showed higher scores for PTSS, state anger, and impacted anger control compared to their colleagues who were not acquainted with the wounded or deceased. Our second finding shows that firefighters who were deployed during the firefighting operation that led to the death of two colleagues showed an impacted outward anger expression and anger control compared to their non-deployed colleagues. Notable limitations of the study include the lack of baseline data before the incident and a possible selection bias among the voluntary participants, as well as uncontrolled confounders such as the participants’ role or exposure intensity during the operation. For future research, we suggest placing a focus on anger trajectories and the interactions between MI, PTSD, and anger among first responders following a distressing incident. The relation between PTSS trajectories and anger trajectories would also be an interesting addition to current research. As part of the CASH-Force project, the study is to be used to adapt special psychological interventions for comparable incidents. The finding that the death or injury of coworkers results in increased scores for PTSS, state anger, and anger control among those who were acquainted with them compared to those who were not is consistent with previous studies. These studies showed an association between PTSS and anger among civilians or veterans who experienced especially distressing life events or combat situations [10,22,23]. Therefore, an association between PTSS and anger should be considered during the post-incident interventions after firefighting operations that led to the death or injury of a known coworker. The fact that the firefighters who were deployed during the operation showed an increase in anger symptoms without higher PTSS compared to their non-deployed colleagues is a novel finding. This finding implies an operation-related variable, which is not necessarily related to PTSD. We assumed this finding to be related to MI. To confirm this assumption, further studies with a focus on the interaction between MI, PTSD, and anger will have to be conducted.

First responders might benefit from regular combined training that includes the realistic simulation of potentially distressing situations as well as mental health education targeting these situations. This training is supposed to give first responders the necessary resources to navigate a real-life scenario more routinely and with a higher awareness of their own well-being [48,49,50]. Additionally, emergency service organizations can decrease the stigma surrounding mental health by promoting their employee’s well-being and encouraging peer support among first responders. An integration of mental health and well-being into everyday working life is supposed to improve the likelihood of first responders asking for and being offered psychological support when needed [48,51]. We recommend periodic monitoring of PTSS and anger in first responders due to their prevalence and their significance as predictors of further comorbidities after distressing incidents [6,7,12,13,14]. By applying such monitoring, emergency services would also generate baseline data that could be used to quantify the impact on first responders after a distressing incident. In the meantime, the study’s results can be applied to similar situations so psychological personnel might be able to foresee possible impacts on those affected. Here, the high impact on any acquaintances of the deceased should be taken into special consideration.

## Figures and Tables

**Table 1:** Testing for differences between the studies’ participants regarding gender, age, years of service, rank, and relationship status.

Variable		Deployed Group (Mean ± SD) or (n (%))	Non-Deployed Group (Mean ± SD) or (n (%))	Test Statistic	*p*-Value	Cohen’s *d*
Age		38.4 ± 10.5	41.0 ± 10.6	T(136) = −1.2	0.226	0.25
Years of service		11.2 ± 9.9	14.6 ± 10.7	T(133) = −1.6	0.110	0.33
Gender	Female	n = 2 (1%) n = 30 (22%)	n = 4 (3%) n = 102 (74%)	χ^2^_(1,n = 138)_ = 0.4	0.547	
	Male					
Rank		-	-	χ^2^_(3,n = 121)_ = 4.8	0.190	
Relationship status		-	-	χ^2^_(1,n = 138)_ = 1.7	0.191	

SD = standard deviation; T = *t*-test; χ^2^ = χ^2^-test.

**Table 2:** Grouping of participants regarding deployment during the operation and acquaintance to the wounded or deceased.

Variable	Deployed Group (n (%))	Non-Deployed Group (n (%))	Test Statistic	*p*-Value
Acquainted to wounded or deceased	n = 15 (11%)	n = 11 (8%)	χ^2^_(n = 138)_ = 27.0	<0.001
Not acquainted to wounded or deceased	n = 17 (12%)	n = 90 (65%)		
Total	n = 32 (23%)	n = 101 (73%)		

SD = standard deviation; χ^2^ = χ^2^-test.

**Table 3:** *t*-test for independent samples to test differences in PCL-5 scores in firefighters who were and were not acquainted with the wounded or deceased.

Variable	N Acquainted (Not Acquainted)	Mean (SD) Acquainted	Mean (SD) Not Acquainted	df	t-Value	*p*-Value	Cohen’s *d*	95% CI of the Difference	
								Lower	Upper
Intrusions	21 (105)	3.38 (3.1)	1.38 (2.16)	24.0	−2.8	0.010	0.75	−3.5	−0.5
Avoidance	21 (107)	1.52 (1.99)	0.5 (0.92)	21.7	−2.3	0.032	0.66	−1.9	−0.1
Negative cognitions	21 (105)	3.67 (4.48)	1.56 (2.58)	22.7	−2.1	0.048	0.58	−4.2	−0.0
Arousal	21 (104)	3.00 (2.74)	2.28 (2.92)	123	−1.0	0.299	0.25	−2.1	0.6
Total-Score	21 (107)	11.57 (10.32)	5.67 (6.98)	23.7	−2.5	0.019	0.67	−10.8	−1.0

CI = Confidence Interval; df = degrees of freedom (with decimal due to lack of homoscedasticity); PCL = Post-traumatic Stress Disorder Checklist; SD = standard deviation.

**Table 4:** *t*-test for independent samples to test differences in STAXI anger control scores in firefighters who were and were not deployed during the operation.

Variable	N Deployed (Not Deployed)	Mean (SD) Deployed	Mean (SD) Not Deployed	df	t-Value	*p*-Value	Cohen’s *d*	95% CI of the Difference	
								Lower	Upper
Anger Control	32 (104)	30.00 (4.75)	27.88 (5.34)	134	2.0	0.046	0.42	0.0	4.2
Anger Control-Out	32 (104)	16.03 (2.81)	14.83 (3.04)	134	2.0	0.048	0.41	0.0	2.4
Anger Control-In	32 (104)	13.97 (3.10)	13.05 (3.35)	134	1.4	0.168	0.28	−0.4	2.2

CI = Confidence Interval; df = degrees of freedom; SD = standard deviation; STAXI-2 = State-Trait Anxiety Inventory-2.

**Table 5:** Linear regression analysis to test for statistically significant influence of acquaintance, deployment, and the interaction between acquaintance * deployment on the dependent variable “Outward anger control”.

Predictor	B	Std. Error	β	t-Value	*p*-Value
Acquaintance	−1.25	0.772	−0.46	−1.6	0.107
Deployment	−1.59	0.741	−0.23	−2.2	0.033
Deployment × Acquaintance	1.15	0.503	0.6	2.3	0.024
$R^{2}$	0.085				
F (3,132)				4.1	0.008

B = unstandardized coefficient; Std. Error = standard error; *β* = standardized coefficient.

## Data Availability

The data collected for this study is not publicly available due to privacy and ethical restrictions. Limited insight may be granted after correspondence with the author and permission of the Bundeswehr Hospital Berlin.
